# The Investigation and Management of Adenomyosis in Women Who Wish to Improve or Preserve Fertility

**DOI:** 10.1155/2018/6832685

**Published:** 2018-03-15

**Authors:** Jin-Jiao Li, Jacqueline P. W. Chung, Sha Wang, Tin-Chiu Li, Hua Duan

**Affiliations:** ^1^Department of Gynecology Minimally Invasive Center, Beijing Obstetrics and Gynecology Hospital, Capital Medical University, Beijing 100006, China; ^2^Department of Obstetrics & Gynaecology, Prince of Wales Hospital, Chinese University of Hong Kong, Shatin, Hong Kong

## Abstract

The management of adenomyosis remains a great challenge to practicing gynaecologists. Until recently, hysterectomy has been the only definitive treatment in women who have completed child bearing. A number of nonsurgical and minimally invasive, fertility-sparing surgical treatment options have recently been developed. This review focuses on three aspects of management, namely, (1) newly introduced nonsurgical treatments; (2) management strategies of reproductive failures associated with adenomyosis; and (3) surgical approaches to the management of cystic adenomyoma.

## 1. Introduction

Adenomyosis is a common benign gynaecological condition but its diagnosis and treatment remain a clinical challenge to physicians. The true incidence of adenomyosis is unknown and the prevalence varies widely due to the lack of a standardized definition and diagnostic criteria. The prevalence from previous retrospective cohort and prospective cohort observational studies is summarized in Tables 1 and 2 [1–9]. Adenomyosis also commonly occurs together with endometriosis. Di Donato et al. [10] showed a prevalence of 21.8% in women undergoing surgery for endometriosis. They also showed an association with parous women, increasing age, dysmenorrhea intensity, and presence of deep infiltrating endometriosis.

Adenomyosis is best defined by Bird in 1972 as “the benign invasion of endometrium into the myometrium, producing a diffusely enlarged uterus which microscopically exhibits ectopic non-neoplastic, endometrial glands and stroma surrounded by the hypertrophic and hyperplastic myometrium” [11].

## 2. Pathogenesis

The exact pathogenesis of adenomyosis remains debatable. The diagnosis of adenomyosis is made when ectopic endometrial implants are found within the myometrium of the uterus. The most common and widely accepted theory involves the downward invagination of the endometrial basalis layer into the myometrium due to either myometrial weakness or altered immunologic activity leading to disruption of the endometrial-myometrial interface, also known as the “junctional zone (JZ)” [12]. Leyendecker et al. [13] showed that uterine auto-traumatisation and the initiation of the mechanism of tissue injury and repair (TIAR) as the primary cause for adenomyosis development based on their method of “visualization” by transvaginal ultrasound (TVS) and cinematographic magnetic resonance imaging (MRI). Their group showed the archimetral compression from the neometral contraction at the onset of menstruation causes high intrauterine pressure, leading to rupture of the archimyometrium at cornual angles. Thus, fragments of the basal endometrium are then detached and deposited into the myometrial wall where they develop into endometriotic cysts. In addition, as the basal stromal cells at the fundo-cornual raphe are chronically over stretched, it initiates the TIAR mechanism and development of an adenomyoma. Other theories include de novo development from embryonic-misplaced pluripotent Mullerian remnants or invagination along the intramyometrial lymphatic system or displaced bone marrow stem cells [14].

## 3. Diagnosis

Histological examination is the gold standard in the diagnosis of adenomyosis, even though the exact histological criteria have not been universally agreed. One accepted criterion is the presence of endometrial tissue more than 2.5 mm below the endomyometrial junction or a JZ thickness of more than 12 mm [15]. The modification of the uterine structure may range from thickening of the JZ of >12 mm to nodular or diffuse lesions involving the entire uterus. Thus, adenomyosis is classified to “diffuse adenomyosis” where endometrial deposits are found dispersed within the myometrium or “focal adenomyoma” where the endometrial deposits are more localized at one site within the uterine wall as a confined lesion [14].

Apart from the findings of these ectopic endometrial tissues within the myometrium, smooth muscle changes like hyperplasia are often found. Ultrastructural differences between smooth muscle cells from adenomyosis and normal uterus were found with myocytes showing cellular hypertrophy, differences in cytoplasmic organelles, nuclear structures, and intercellular junctions [15]. The myocytes in adenomyosis also lack the cyclical changes present in myocytes of the normal uterus [16].

## 4. Cystic Adenomyoma

Rarely, adenomyosis may present as a cystic lesion lined with endometrial tissue and surrounded by myometrial tissue when it is called “cystic adenomyoma.” Juvenile cystic adenomyoma (JCA) is a subgroup of cystic adenomyoma that commonly occurs in adolescents or women < 30 years of age and is not associated with diffuse adenomyosis. Takeuchi et al. [17] proposed the following diagnostic features of juvenile cystic adenomyoma (JCA): (1) age < 30 years; (2) cystic lesion of >1 cm in diameter independent of the uterine lumen and covered by hypertrophic myometrium on diagnostic images; and (3) association with severe dysmenorrhea. They found that laparoscopic excision of the lesion demonstrated significant improvement of dysmenorrhea in these cases.

## 5. Presentations

The classic presentation of adenomyosis is heavy, painful menstrual bleeding, typically occurring in multiparous women between 40 and 50 years of age [14]. Heavy menstrual bleeding is present in up to 40–60% of patients, which may be due to the enlarged endometrial surface area or the increased vascularity of the endometrium [14]. Dysmenorrhea occurs in 15–30% of patients, which may be related to the swelling of endometrial tissue within the myometrium or increased production of prostaglandin within the myometrium [18]. Both the amount of bleeding and degree of pain were shown to be significantly correlated with the degree of myometrial invasion [18]. Other presenting features include chronic pelvic pain, dyspareunia, and the finding of an enlarged uterus in an asymptomatic subject. Women with adenomyosis had been shown to have a decreased quality of life [19], up to 33% of patients may be asymptomatic, and the diagnosis of up to 30% of patients was only made by histology following a hysterectomy [20].

There is also increasing evidence to show an association between infertility and adenomyosis [21]. Several mechanisms may be involved, including impairment of sperm transport [7], aberrant uterine contractility [22], alterations of adhesion molecules, cell proliferation, apoptosis, and free radical metabolism [15, 23]. Adenomyosis is also speculated to be a cause of recurrent implantation failure during IVF treatment [24].

## 6. Investigation

### 6.1. Two-Dimensional Ultrasound (USG)

Two-dimensional (2D) transabdominal USG may reveal uterine enlargement or asymmetric thickening of the anterior and posterior myometrial walls. However, transabdominal USG is often not accurate enough in diagnosing adenomyosis as it fails to provide sufficient image resolution for visualization of the myometrium. Therefore, 2D transvaginal USG is often the first-line investigation. In a review performed by Reinhold et al., it was shown that transvaginal USG had a sensitivity of 80–86%, specificity of 50–96%, and an overall accuracy of 68–86% in diagnosing diffuse adenomyosis [25].

USG features of adenomyosis include the presence of three or more sonographic criteria: heterogeneity, increased echogenicity, decreased echogenicity, and anechoic lacunae or myometrial cysts [26]. In contrast to uterine fibroids, adenomyoma has a more elliptical shaped lesion with poorly defined borders, no calcifications, or edge shadowing. In doubtful cases, Doppler sonography may be helpful in that blood vessels in the case of adenomyoma usually follow their normal vertical course in the myometrial areas while in the case of uterine fibroid, blood vessels are usually located in the periphery [27].

Sonographic diagnosis of adenomyosis is not always easy but the consensus statement and recommendation published by the MUSA (Morphological Uterus Sonographic Assessment) group on how sonographic features of adenomyosis should be described and measured should help to improve the diagnostic accuracy [28].

### 6.2. Three-Dimensional Ultrasound

Three-dimensional (3D) USG improves diagnostic accuracy of adenomyosis as it allows better imaging of the JZ [29]. The JZ is often visible as a hypoechogenic subendometrial halo which is composed of longitudinal and circular closely packed smooth muscle fibers. Upon 3D USG, adenomyosis is characterized by a thickened or irregular JZ [30]. Ahmadi and Haghighi showed the accuracy of 3D transvaginal USG in the diagnosis of adenomyosis to be 80% and a positive predictive value of 95% based on the detection of an irregular JZ on coronal plane [31]. Exacoustos et al. [30] analyzed a total of 72 premenopausal patients with 2D and 3D transvaginal USG before hysterectomy. In the study, the histological prevalence of adenomyosis was 44.4%. Their group agrees that the coronal section of the uterus obtained by 3D transvaginal USG allows accurate evaluation and measurement of the JZ and its alteration shows good diagnostic accuracy for adenomyosis. They showed that the presence of myometrial cysts was the most specific 2D transvaginal USG feature with specificity of 98% and accuracy of 78% while heterogeneous myometrium was the most sensitive feature with a sensitivity of 88% and accuracy of 75%. As for 3D transvaginal USG, with a JZ difference of more than or equal to 4 mm, JZ infiltration and distortion had a high sensitivity of 88% and the best accuracy of 85% and 82%, respectively. The overall accuracy of diagnosing adenomyosis for 2D and 3D transvaginal USG was 83% and 89%, sensitivity was 75% and 91%, specificity was 90% and 88%, positive predictive value was 86% and 85%, and negative predictive value was 82% and 92%, respectively. 3D USG also has the advantage of allowing storage of the images with subsequent offline manipulation and interpretation.

### 6.3. Magnetic Resonance Imaging

Magnetic resonance imaging (MRI) is the gold standard imaging modality for assessing the JZ in the evaluation of adenomyosis [32]. The common features of adenomyosis on MRI include (1) thickening of the JZ, JZ thickness ≥ 12 mm, or irregular junctional thickness with a difference of >5 mm between the maximum thickness and the minimum thickness, (2) an ill-defined area of low signal intensity in the myometrium on T2-weighted MR images, and (3) islands of ectopic endometrial tissue identified as punctate foci of high signal intensity on T1-weighted image [32–34]. However, MRI is expensive and may not be readily available in every unit. Moreover, Reinhold et al. [33] prospectively studied 119 patients undergoing hysterectomy and compared findings between TVS and MRI. The study showed that there was no significant difference in sensitivity and specificity between the two groups. Champaneria et al. [34] also performed a systematic review comparing test accuracy between USG and MRI for the diagnosis of adenomyosis. Their study findings are summarized in Table 3. They agreed that both TVS and MRI show high levels of accuracy for the noninvasive diagnosis of adenomyosis. However, we believe MRI may be particularly useful in the assessment of focal adenomyoma and provides important information on whether surgery should proceed.

### 6.4. Shear Wave Elastography

A recent study also showed that using Aixplorer (Supersonic Imagine, France) scanner with application of shear wave elastography during transvaginal scanning may improve diagnostic accuracy of adenomyosis [35]. This study found that adenomyosis was associated with a significant increase of the myometrial stiffness estimated with shear wave elastography. Further studies are required to verify the clinical usefulness of such an approach.

### 6.5. Hysterosalpingography

Hysterosalpingography is seldom used to diagnose adenomyosis. However, in patients undergoing infertility assessment, the occasional finding of spiculations measuring 1–4 mm in length, arising from the endometrium towards the myometrium, or a uterus with the “tuba erecta” finding may be suggestive of adenomyosis [36].

### 6.6. Hysteroscopy

Several hysteroscopic appearances have been found to be associated with adenomyosis, including irregular endometrium with endometrial defects or superficial openings, hypervascularization, strawberry pattern, or cystic haemorrhagic lesions [37]. Nevertheless, there is limited data available on the diagnostic accuracy of these various features.

### 6.7. Hysteroscopic and Laparoscopic Myometrial Biopsy

In 1992, McCausland [38] showed that myometrial biopsy is helpful to diagnose adenomyosis. The study found that the depth of adenomyosis was correlated with the severity of menorrhagia. Of the 90 patients studied, 50 patients had normal hysteroscopy in which 55% of them had significant adenomyosis (greater than 1 mm) when compared to controls (0.8 mm). In that study, it was suggested that minimal adenomyosis may be treated definitively by endometrial ablation while deep adenomyosis should be treated by hysterectomy. They also showed that endometrial glands left under a scar could not only bleed and cause pain but also have malignant potential. The authors suggested routine myometrial biopsy at the time of operative hysterectomy should be considered. However, Darwish et al. [39] showed hysteroscopic myometrial biopsies using rigid biopsy forceps to be inadequate and did not recommend its use. Popp et al. [40] showed that the sensitivity of a single myometrial biopsy in diagnosing adenomyosis ranged from 8 to 18.7%, while the specificity was 100% among 680 biopsy specimens in 68 surgically removed uterus using automatic cutting needle sampling. Gordts et al. [41] recommended the use of hysteroscopic guided biopsy for the diagnosis of adenomyosis using a new device, the Utero-Spirotome. It can also be used under ultrasound guidance to get access to small cystic adenomyoma lesions.

### 6.8. Laparoscopic Myometrial Biopsy

In a prospective, nonrandomized study conducted by Jeng et al. [42] evaluating 100 patients with clinical signs and symptoms strongly suggestive of adenomyosis, the sensitivity of myometrial biopsy was 98% and the specificity 100%; the positive predictive value was 100% and the negative predictive value 80%, which were superior to those of transvaginal sonography, serum CA-125 determination, or the combination of both. The group suggested that laparoscopy-guided myometrial biopsy is a valuable tool in the diagnosis of diffuse adenomyosis in women presenting with infertility, dysmenorrhea, or chronic pelvic pain.

## 7. Management

As in the case of endometriosis, the management strategy of adenomyosis depends primarily on the presenting symptom and whether it is associated with reproductive failure.

### 7.1. Management of Menstrual Symptoms

#### 7.1.1. Medical Treatment

Medical treatment for adenomyosis is similar to those given for endometriosis. Apart from symptomatic relief, hormonal treatment mainly works by inhibition of ovulation, cessation of menses, improving the hormonal milieu, and causing decidualization of the endometrial deposits.

*Analgesic*. Nonsteroidal anti-inflammatory drugs (NSAIDs) work by inhibiting the cyclooxygenase (COX-1 and COX-2) and decreasing the production of prostaglandins. NSAIDs have been proved to be effective in treatment of primary dysmenorrhea by Gambone et al. [43]. It is usually the first-line treatment for symptomatic pain relief for adenomyosis.

*Oral Contraceptive Pills (OCPs).* Combined oral contraceptive pills work by inhibiting ovulation by suppressing the release of gonadotrophins. Many studies have shown that they are effective in the treatment of dysmenorrhea. A prospective observational trial showed that continuous low-dose OCP were more effective than cyclical low-dose OCP in controlling symptoms in patients after surgical treatment for endometriosis [44]. Mansouri et al. [45] have shown regression of adenomyosis on MRI after using oral contraceptive pills for 3 years in adolescents with adenomyosis presenting with chronic pelvic pain.

*Danazol.* Danazol is an isoxazol derivative of 12 alpha-ethinyl testosterone. It causes a hypogonadic state and thus is widely used for treatment of endometriosis and abnormal uterine bleeding [46]. However, data on its use in adenomyosis remains limited. This may be due to its unwanted adverse effects after systemic treatment. In 2000, Igarashi et al. [47] reported a novel conservative medical therapy for uterine adenomyosis with a danazol-loaded intrauterine device in 14 women. During insertion of the danazol-loaded IUD, there was complete remission of dysmenorrhea in 9 patients, reduction in 4, and no change in 1 patient. There was complete remission of hypermenorrhea in 12 patients and no change in 2. Nine out of 14 patients also showed reduction in the maximum thickness of the myometrium as measured by MRI. However, further studies are required to confirm the clinical usefulness of the treatment.

*Dienogest*. Dienogest is a selective synthetic oral progestin that combines the pharmacological properties of 17-alpha-progesterone and 19 nor-progesterone with pronounced local effect on endometrial tissue. Dienogest has been shown to be effective in the treatment of endometriosis associated pelvic pain. A prospective clinical trial has shown dienogest to be a valuable alternative to depot triptorelin acetate for treatment of premenopausal pelvic pains in women with uterine adenomyosis. The study included a total of 41 patients with adenomyosis with pelvic pain and menorrhagia. The patients were allocated to receive oral dienogest (2 mg/day) or triptorelin acetate (3.75 mg/4 weeks) for 16 weeks. Both treatments were highly effective in treatment of dysmenorrhea, dyspareunia, and chronic pelvic pain associated with adenomyosis, although triptorelin acetate appeared superior to dienogest in controlling menorrhagia [48].

*Levonorgestrel-Releasing Intrauterine Device (LNG-IUD)*. LNG-IUD is an intrauterine device, which release 20 micrograms of levonorgestrel per day. It has been shown to be an effective treatment for abnormal uterine bleeding. LNG-IUD acts locally and causes decidualization of the endometrium and adenomyotic deposits. LNG-IUD alleviates dysmenorrhea by improving uterine contractility and reducing local prostaglandin production within the endometrium. LNG-IUD appears to be an effective method in relieving dysmenorrhea associated with adenomyosis [49] and more effective than the combined OC pill [50], improved the quality of life [19], and appears to be a promising alternative treatment to hysterectomy.

LNG-IUD may be used in conjunction with other treatment modalities such as GnRH analogue [51] or transcervical resection of the endometrium (TCRE) [52]. In the latter study, it was found that TCRE combined with LNG-IUD was more effective in reducing menstrual flow compared with the LNG-IUD alone although there was no significant difference in the amount of pain reduction between the two treatment strategies.

*GnRH Agonists*. GnRH agonists are effective in alleviating dysmenorrhea and relieving menorrhagia associated with adenomyosis [53]. However, due to the undesirable climacteric side effects and risk of osteoporosis, treatment with GnRH agonists is usually restricted to a short duration of 3–6 months although the duration of use may be extended if add-back estrogen therapy is employed [54]. Discontinuation of treatment usually leads to regrowth of the lesions and recurrence of symptoms.

*Selective Estrogen Receptor Modulator (SERM)*. Selective estrogen receptor modulators like tamoxifen or raloxifene have been tried in the treatment of endometriosis [54] based on observations that SERMs may reduce endometriosis lesion in mouse [55]; however, their value in the treatment of adenomyoma has not been formally explored.

*Aromatase Inhibitors*. Like endometriosis, adenomyotic deposits are estrogen-dependent. Aromatase inhibitors inhibit the conversion of estrogen from androgens, thereby lowering the synthesis of estrogen. A prospective randomized controlled study found that the efficacy of aromatase inhibitors (letrozole 2.5 mg/day) in reducing the volume of adenomyoma as well as improving adenomyosis symptoms was similar to that of GnRH agonists (goserelin 3.6 mg/month) [56]. Kimura et al. also reported on the combined use of aromatase inhibitors with GnRH agonist with good results in a 34-year-old woman with severe uterine adenomyosis who wished to preserve fertility [57]. They found a reduction in uterine volume of 60% after 8 weeks of treatment as determined by magnetic resonance imaging and ultrasound.

*Ulipristal Acetate.* Ulipristal acetate (UPA) is a potent selective progesterone receptor modulator. There is good evidence to suggest that it can be used to shrink fibroid and control menorrhagia [58, 59]. It is possible that it may be similarly effective in the treatment of adenomyoma but literature data is lacking.

*Antiplatelet Therapy.* There is new evidence to suggest a role of antiplatelet therapy in treating adenomyosis. Emerging evidence suggests that endometriotic lesions are wounds undergoing repeated tissue injury and repair (ReTIAR), and platelets induce epithelial-mesenchymal transition (EMT) and fibroblast-to-myofibroblast transdifferentiation (FMT), leading ultimately to fibrosis. Adenomyotic lesions are thought to have similar pathogenesis to that of endometriosis. A recent study in mice suggests that antiplatelet treatment may suppress myometrial infiltration, improve generalized hyperalgesia, and reduce uterine hyperactivity [60].

#### 7.1.2. Uterine Artery Embolization

Uterine artery embolization (UAE) has been used to treat symptomatic fibroids since the 1990s. There is increasing evidence to suggest that it is also effective in the treatment of management of adenomyosis. In a review of 15 studies including 511 women with adenomyosis, Popovic et al. found [61] significant clinical and symptomatic improvement in seventy-five percent of subjects at short- and long-term follow-up. A recent retrospective observational study of 252 patients who underwent UAE with up to five years of follow-up showed that improvement in dysmenorrhea and menorrhagia are more likely to occur in vascular lesions [62].

#### 7.1.3. High Intensity Focused Ultrasound

High intensity focused ultrasound (HIFU) is another nonsurgical treatment for uterine fibroids that focuses high intensity ultrasound in the target lesion causing coagulative necrosis and shrinkage of the lesion. Both MRI and USG can be used for guidance for the procedure. MRI has better real time thermal mapping during the HIFU treatment. Yet, ultrasound guided HIFU is less costly and offers real time anatomic monitoring imaging and a grey scale change during treatment represents a reliable indicator in treatment response. It is effective in both focal and diffuse lesions [63, 64]. Ultrasound guided HIFU was shown to be technically successful in up to 94.6% of patients in a review of 2549 patients among 10 different centers with symptomatic adenomyosis [65].

#### 7.1.4. Endomyometrial Ablation or Resection

There is limited report on the use of laparoscopic or hysteroscopic endometrial in treating adenomyosis in the literature. The success rate of myometrial electrocoagulation ranges from 55 to 70% as reported [66]. Wood [67] reported success in 4 out of 7 patients who underwent myometrial electrocoagulation, while Phillips et al. [68] had 7 out of 10 patients with symptomatic adenomyosis diagnosed by MRI treated with laparoscopic bipolar coagulation, having significant reduction or resolution of dysmenorrhea or heavy menstrual bleeding.

#### 7.1.5. Hysterectomy

Hysterectomy is the definitive treatment option for intractable symptomatic adenomyosis when medical or other conservative treatments have failed to control the symptoms. Patients undergoing hysterectomy for adenomyosis should be advised of an increased risk of bladder injury and persistent pelvic pain. Furuhashi et al. [69] reviewed 1246 vaginal hysterectomies and found that patients undergoing vaginal hysterectomy for adenomyosis have increased risk of bladder injury compared with those performed for leiomyoma (2.3% versus 0.7%). It may be a result of difficulty in identifying the supravaginal septum and the vesicovaginal or vesicocervical planes. Several studies have reported on persistent pelvic pain after hysterectomy for adenomyosis [70]. Once a decision to proceed with hysterectomy has been made, the possibility of oophorectomy should be discussed. In general, it is not considered necessary to routinely remove the ovaries in premenopausal women [71, 72], but it may be indicated in women who suffer from cyclical symptoms, with concomitant ovarian endometriosis, or who are considered to have an increased risk of developing ovarian cancer, including those with a family history of the condition. Interestingly, a recent population-based study by Kok et al. [73] suggested that the risk of developing ovarian cancer in women with newly diagnosed adenomyosis is increased by 4-5-fold. If the finding is confirmed, there is a strong case to consider prophylactic oophorectomy at the time of hysterectomy for adenomyosis in premenopausal women.

### 7.2. Reproductive Failure

Several studies have shown that adenomyosis is associated with a negative impact on the success rate of IVF. In a recent meta-analysis conducted by Vercellini et al. [74], adenomyosis was associated with a 28% reduction in the likelihood of a clinical pregnancy in infertile women who underwent IVF/ICSI with autologous oocytes. Patients with adenomyosis were found to have higher chances of miscarriage, independent of oocyte or embryo quality. Thalluri and Tremellen [75] also showed that the adenomyosis was associated with a significant reduction in successful implantation of good-quality embryos in patients undergoing IVF treatment (viable clinical pregnancy rate 23.6% versus 44.6% among those who did not have adenomyosis, *P* = 0.017).

Puente et al. [76] performed a cross-sectional study of 1015 patients prior to assisted conception treatment. They found that the prevalence of adenomyosis was 24.4% in women aged ≥40 years and 22% in women aged ≤40 years. The prevalence of adenomyosis was found to be higher in those with recurrent pregnancy loss (38.2%) and previous ART failure (34.7%) when compared with those who did not (22.3% and  24.4%, respectively). They also found that 4 out of 5 patients had the diagnosis missed in earlier transvaginal ultrasonography.

The use of short-term GnRH agonists to shrink the size of the adenomyosis lesion has been shown to improve conception rate within 6 months of cessation of GnRH agonist therapy [77, 78].

In women with adenomyosis planning to undergo IVF treatment, the following management strategies should be considered.

#### 7.2.1. GnRH Analogue Therapy before In Vitro Fertilization

Several studies have shown that pretreatment with GnRH analogue before IVF treatment improved pregnancy outcome. Zhou et al. [79] analyzed the clinical efficacy of leuprorelin acetate in treatment of uterine adenomyosis with infertility. They found that, after 2–6 months of leuprorelin acetate therapy, the mean uterine volume was significantly reduced from 180 ± 73 cm^3^ to 86 ± 67 cm^3^, leading to an improvement in embryo implantation and clinical pregnancy rates.

#### 7.2.2. Stimulation Protocol

In women without pre-IVF GnRH analogue therapy as described above, long GnRH analogue protocol should be considered as it helps to induce decidualization of the adenomyotic deposits rendering the disease inactive. Tao et al. [80] showed that GnRH antagonist protocol appears to be inferior to GnRH agonist long protocol cycle, and the latter appeared to be associated with increased pregnancy and decreased miscarriage rates.

#### 7.2.3. Two-Staged In Vitro Fertilization

In women with adenomyosis, a two-staged in vitro fertilization could be considered. Patients can undergo ovarian stimulation, oocyte retrieval, and fertilization followed by frozen-thawed embryo transfer (FET) at a later stage. Prior to the FET, GnRH analogue suppression therapy for 3 months or so leads to shrinkage of the adenomyosis. FET in the first HRT cycle following GnRH analogue suppression therapy, before the adenomyosis lesion regrows to its pretreatment size and exerts its adverse impact on implantation, may improve the result.

#### 7.2.4. Mock Embryo Transfer

Performing a mock embryo transfer is desirable in women with adenomyosis, as it may help to assess the uterine cavity length and position, choose the correct transfer catheter, and alert the clinicians any extra precautions (e.g., use of tenaculum or cervical dilatation). Mock embryo transfer is particularly desirable in those with an enlarged uterus or distorted uterine cavity.

#### 7.2.5. Single Embryo Transfer

Adenomyosis has been reported to be associated with increased incidence of preterm delivery, preeclampsia, and second trimester miscarriage when compared with the control group [81]. Consequently, multiple pregnancies should be avoided and so single embryo transfer should be advised. Women who had adenomyomectomy prior to IVF should also be advised to have SET to avoid multiple pregnancy with a view to minimize the risk of scar rupture.

#### 7.2.6. HRT Protocol in Frozen-Thawed Embryo Transfer (FET) Cycle

GnRH agonist pretreatment to suppress the pituitary ovarian axis prior to hormone replacement therapy to prepare the endometrium in FET cycles appeared to improve the outcome compared with hormone replacement therapy without downregulation. In a study including 339 patients with adenomyosis, 194 received long-term GnRH agonist plus HRT (downregulation + HRT) and 145 with HRT alone. The clinical pregnancy, implantation, and ongoing pregnancy rates in the downregulation and HRT group were significantly higher than that of the HRT alone group, being 51.35% versus 24.83%, 32.56% versus 16.07%, and 48.91% versus 21.38%, respectively [82].

#### 7.2.7. Uterine Contractility and Atosiban Therapy

Several functional studies showed that excessive uterine contractility (>5 contractions per minute) has been demonstrated in approximately 30% of patients undergoing embryo transfer and this may have a significant adverse impact on subsequent embryo implantation and clinical pregnancy rates [83]. The incidence of abnormal contractility appeared to be higher in women with adenomyosis [84] which may in part explain the higher incidence of reproductive failure observed in this group of women. Although recent evidence suggests that the routine use of atosiban therapy does not improve the outcome [85], it is possible that the use of atosiban in a selected group of women with aberrant uterine contractions during embryo transfer may improve the outcome. Ideally, women with adenomyosis should be screened for abnormal uterine contractions during ET; if the results are abnormal atosiban therapy should be discussed; alternatively, the possibility of empirical atosiban therapy in women with adenomyosis and recurrent implantation failure could be considered.

#### 7.2.8. Recurrent Implantation Failure

Recurrent implantation failure is diagnosed when there is failure to achieve a clinical pregnancy after transfer of at least four good-quality embryos in a minimum of three fresh or frozen cycles in a woman under the age of 40 years [86]. It is known that adenomyosis is associated with recurrent implantation failure [24]. Women with recurrent implantation failure should be offered 3D scan or MRI to establish if there is adenomyosis; if adenomyosis is present, the above management strategies should be adopted to improve the outcome.

#### 7.2.9. Uterine Sparing Conservative Surgery

Surgery is seldom required for women prior to IVF treatment, the indication being (1) well-defined adenomyoma more than 5 cm and (2) recurrent miscarriage or recurrent implantation failure after IVF. A retrospective cohort study performed by Kishi et al. [87] involving 102 women showed that laparoscopic adenomyomectomy was beneficial for women who experienced IVF treatment failures if they were <39 years old but not for patients aged 40 years or more. No benefit of uterine sparing surgery is seen for those older patients aged 40 or above. Grimbizis et al. [88] reviewed the current literature and described three main categories of uterine sparing surgical treatment, including complete excision by adenomyomectomy; cystectomy or partial excision cytoreductive surgery; and nonexcisional techniques including uterine artery ligation, electrocoagulation of myometrium, resection, and ablation. The review concluded that uterine sparing treatment of adenomyosis appears feasible and effective. After complete excision, the dysmenorrhea reduction, menorrhagia control, and pregnancy rate were 82.0%, 68.8%, and 60.5%, respectively. After partial excision, the dysmenorrhea reduction rate was similar at 81.8%, although menorrhagia control and pregnancy rate were slightly reduced to 50.0% and 46.9%, respectively.

### 7.3. Hysteroscopic Surgery

Just as it is now possible to remove intramural myoma with refined hysteroscopic techniques, hysteroscopic adenomyomectomy may also be possible in selected cases, especially when the adenomyoma is <5 cm or when it protrudes into the uterine cavity. However, hysteroscopic adenomyomectomy should always be carried out under USG guidance. A minimal safety margin of 5 mm between the serosa and adenomyoma is considered necessary to avoid the risk of uterine perforation although the safety margin may sometimes increase after part of the lesion has been removed and the uterine contractions which follow help to push the adenomyoma further towards the cavity. Pretreatment with 3-month course of GnRH agonist beforehand can help reduce the vascularity and bleeding during the operation. Sometimes, it may also help to push the adenomyoma towards the uterine cavity due to the reduction of uterine volume.

The location of the adenomyoma should be clearly defined before the start of the procedure. Using a lower perfusing pressure, say at 40 mmHg instead of the usual 90–100 mmHg, may allow a slight bulge of the adenomyoma into the cavity to be visualized. Vasopressin, a potent vasoconstrictor, may be injected into the uterus by using an oocyte retrieval needle [89] to result in contraction of the uterus and reduce bleeding. Afterwards the endometrium and the myometrium overlying the adenomyoma can be incised using a cutting loop or needle or dissected with the use of a pair of scissors, following which the adenomyoma is removed by the cutting loop or a pair of grasping forceps coupled with twisting actions, separating it from the underlying myometrium. The latter step may be achieved with the use of Hysteroscopy Endo-Operative System (HEOS) [90], which allows both mechanical and electrosurgical instruments to be used. Complete removal of the adenomyoma may be difficult. A repeat surgical procedure may be required from time to time.

**Figure 1:**
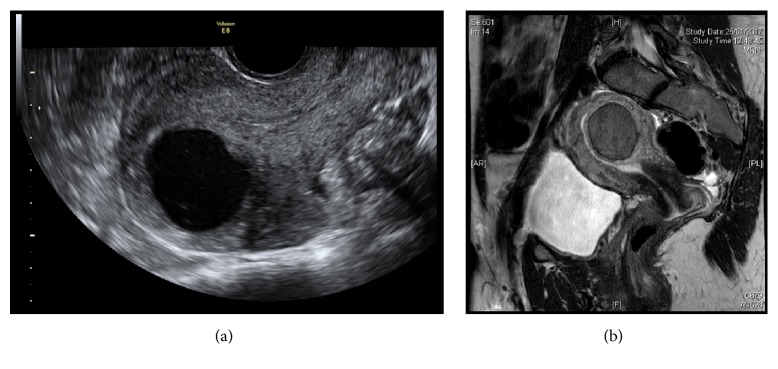
(a) Ultrasound and (b) MRI appearance of a cystic adenomyoma.

**Figure 2:**
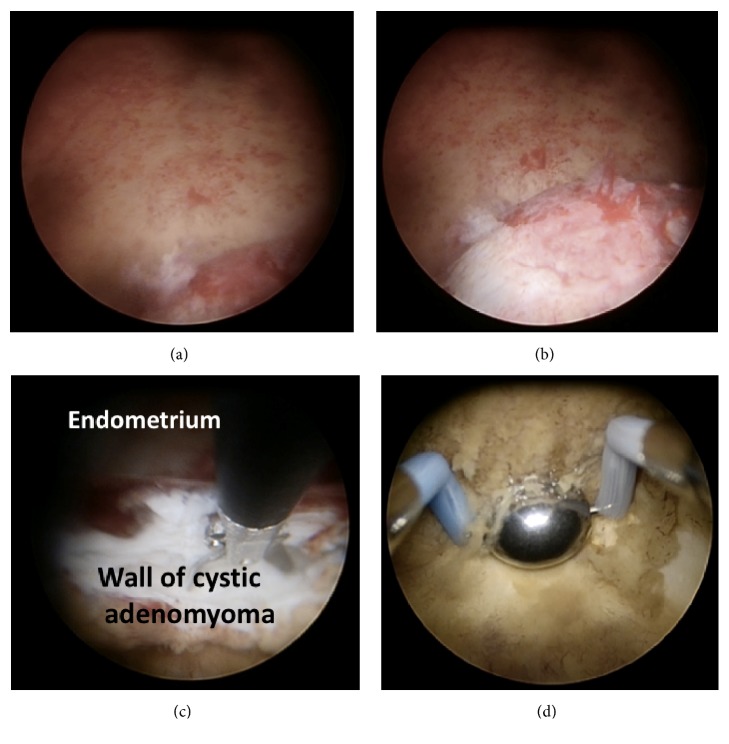
(a) Hysteroscopic view at high perfusion pressure. (b) Hysteroscopic view at low perfusion pressure with bulging of cystic adenoma seen. (c) Hysteroscopic dissection of cystic adenomyoma wall away from endometrium. (d) Roller ball ablation of adenomyotic deposits.

Cystic adenomyoma is a special category of adenomyoma. Figures 1(a) and 1(b) show the ultrasound and MRI appearance of a cystic adenomyoma. At the beginning of the hysteroscopic operation, the adenomyoma did not appear to bulge into the cavity (Figure 2(a)), but upon lowering the perfusing pressure, the cystic adenomyoma was seen bulging into the cavity (Figure 2(b)), which permits the precise location of the lesion to be identified. In this particular case, a longitudinal incision was made over the adenomyoma, draining a large amount of blood clots from the cystic adenomyoma. In this case, initial attempts to dissect the cystic adenomyoma away from the myometrium (Figure 2(c)) had to be abandoned because the lesion was too firmly adherent to the myometrium, without a well-defined cleavage plane, in contrast to the situation of a myoma. Consequently, the cyst wall, including the yellow-brown deposits representing the ectopic endometriotic deposits (Figure 2(d)), was ablated under ultrasound guidance with the use of a roller ball diathermy.

## 8. Conclusion

Many treatment modalities are now available for the treatment of adenomyosis. The management plan ought to be individualized, depending on the presenting symptom and the desire to achieve a successful pregnancy. Recent development in various nonsurgical and surgical options has significantly improved the prospect of a successful treatment in women wishing to conceive again.

## Figures and Tables

**Table 1:** Prevalence of adenomyosis after hysterectomy specimens for various gynaecological conditions (from retrospective cohort studies).

Study	Vercellini et al. 1995 [1]	Vavilis et al. 1997 [2]	Seidman and Kjerulff1996 [3]	Parazzini et al. 1997 [4]	Bergholt et al. 2001 [5]
Number of cases (*n*)	1334	594	1252	707	549
Adenomyosis (%)	25	20	12–58	21	10–18
Uterine fibroid	23	21		15	
Genital prolapse	26	26		30	
Ovarian cyst	21	18		30	
Cervical cancer	19	18		25	
Endometrial cancer	28	16			
Ovarian cancer	28	21			

**Table 2:** Prevalence of adenomyosis from previous prospective cohort observational studies.

Study	Number of patients (*n*)	Study characteristics	Diagnostic modality	Definition of adenomyosis	Prevalence%
de Souza et al. 1995 [6]	26	Infertility patients presenting with dysmenorrhea or menorrhagia, all had laparoscopy performed	MRI	Focal adenomyoma: ill-defined lesions within the myometrium	54
				Diffuse adenomyosis: diffuse or irregular JZ thickening	

Kunz et al. 2005 [7]	227	Study group (*n* = 160): infertility patients with laparoscopy done showing endometriosis	MRI	Focal adenomyoma: expansions of variable shape and size that did not extend over the whole length of the uterine cavity Diffuse adenomyosis: expansion of anterior or posterior JZ	79 90 28
		Study subgroup: presence of endometriosis, <36 years old with fertile partners			
		Control group (*n* = 67): infertility patients with no endometriosis or other pelvic disorder on laparoscopy			

Kissler et al. 2008 [8]	70	Patients with severe dysmenorrhea with laparoscopy performed	MRI	Maximal thickness >8 mm or greater on T2 weighted images	53 87
		Group I: patients with dysmenorrhea < 11 years			
		Group II: patients with dysmenorrhea > 11 years			

Naftalin et al. 2012 [9]	985	Consecutive patients attending the general gynaecology clinic	TVS	Asymmetrical myometrial thickening not caused by presence of fibroids, parallel shadowing, linear striations, myometrial cysts, hyperechoic islands, adenomyoma, and irregular JZ	21

MRI: magnetic resonance imaging; TVS: transvaginal ultrasound scan; JZ: junctional zone.

**Table 3:** Accuracy of TVS and MRI for the noninvasive diagnosis of adenomyosis.

	TVS	MRI
Sensitivity	72%	77%
Specificity	81%	89%
Positive likelihood ratio	3.7	6.5
Negative likelihood ratio	0.3	0.2

TVS: transvaginal ultrasound scan; MRI: magnetic resonance imaging.
